# Effects of 3FTx Protein Fraction from *Naja ashei* Venom on the Model and Native Membranes: Recognition and Implications for the Mechanisms of Toxicity

**DOI:** 10.3390/molecules26082164

**Published:** 2021-04-09

**Authors:** Barbara Dyba, Elżbieta Rudolphi-Szydło, Anna Barbasz, Agnieszka Czyżowska, Konrad Kamil Hus, Vladimír Petrilla, Monika Petrillová, Jaroslav Legáth, Aleksandra Bocian

**Affiliations:** 1 Department of Biochemistry and Biophysics, Faculty of Biology, Pedagogical University of Cracow, Podchorążych 2, 30-084 Cracow, Poland; barbara.dyba@up.krakow.pl (B.D.); elzbieta.rudolphi-szydlo@up.krakow.pl (E.R.-S.); anna.barbasz@up.krakow.pl (A.B.); czyzowskaa@gmail.com (A.C.); 2 Department of Biotechnology and Bioinformatics, Faculty of Chemistry, Rzeszow University of Technology, Powstańców Warszawy 6, 35-959 Rzeszów, Poland; k.hus@prz.edu.pl (K.K.H.); jaroslav.legath@uvlf.sk (J.L.); 3 Department of Biology and Physiology, University of Veterinary Medicine and Pharmacy in Košice, Komenského 73, 041-81 Košice, Slovakia; petrillav@gmail.com; 4 Zoological Department, Zoological Garden Košice, Široká 31, 040 06 Košice-Kavečany, Slovakia; 5 Department of General Competencies, University of Veterinary Medicine and Pharmacy in Košice, Komenského 73, 041-81 Kosice, Slovakia; monika.petrillova@uvlf.sk; 6 Department of Pharmacology and Toxicology, University of Veterinary Medicine and Pharmacy, Komenského 73, 041-81 Košice, Slovakia

**Keywords:** venom, three-finger toxins, model membranes, Langmuir monolayers, human cells, cytotoxicity

## Abstract

Three-finger toxins are naturally occurring proteins in Elapidae snake venoms. Nowadays, they are gaining popularity because of their therapeutic potential. On the other hand, these proteins may cause undesirable reactions inside the body′s cells. A full assessment of the safety of *Naja ashei* venom components for human cell application is still unknown. The aim of the study was to determine the effect of the exogenous application of three-finger toxins on the cells of monocytes (U-937) and promyelocytes (HL-60), with particular emphasis on the modification of their membranes under the influence of various doses of 3FTx protein fraction (0–120 ng/mL). The fraction exhibiting the highest proportion of 3FTx proteins after size exclusion chromatography (SEC) separation was used in the experiments. The structural response of cell membranes was described on the basis of single-component and multi-component Langmuir monolayers that mimicked the native membranes. The results show that the mechanism of protein–lipid interactions depends on both the presence of lipid polar parts (especially zwitterionic type of lipids) and the degree of membrane saturation (the greatest-for unsaturated lipids). The biochemical indicators reflecting the tested cells (MDA, LDH, cell survival, induction of inflammation, LD50) proved the results that were obtained for the model.

## 1. Introduction

Snake venoms are mixtures composed of a large group of biologically active proteins [1,2]. Neurotoxins (NT), cardiotoxins (or cytotoxins, CT), and phospholipase A_2_ (PLA_2_) are the three main groups of components that are responsible for the toxicity of Elapidae snake venoms [3,4], wherein NTs and CTs belong to the family of non-enzymatic proteins named three-finger toxins (3FTxs) [5,6]. 3FTxs are the most common toxins found in Elapidae venoms, including *Naja ashei* venom [7,8]. The species of this snake was designated in 2007 [9], which means that the structural and biochemical analysis of its venom’s effects on bioactive components on human cells has not been fully analyzed. Interestingly, nowadays, the effects of various venom components, collected from snakes also belonging to the *Naja* genus, on tumor cells [10,11], normal cells [12], bacteria [13], fungi [14], and membranes [15] is being intensively investigated. Proteins belonging to the 3FTx superfamily consist of 60–74 amino acid residues. The group is named for its common structure consisting of three β-strand loops connected to a central core containing four conserved disulfide bonds [16].

All 3FTxs have a similar protein folding structure, but their biological activity can differ significantly [17,18,19]. It may depend not only on the nature of the toxin, but also on various components of the protein-lipid membranes [17].

In the presented scientific papers, neurotoxins (NT) belonging to the 3FTxs act through specific interactions with protein receptors, though no specific protein target for CTxs (cardiotoxins/cytotoxins) has been found. Additionally, unlike NT, CTs are amphiphilic and can cause cytotoxic effects to variety of cells. Some representatives of the CTx group occur in *Naja ashei* venom, e.g., CTxM1, CTxM4, and CTxM5 isoforms [7,8]. Different research studies indicate that this group of proteins has the ability to interact with lipid membranes. The main biological targets of these cytotoxins are damage to the structure of membranes, interaction with selected phospholipids, and penetration/insertion of protein loops in hydrophobic bilayer parts [15,20,21]. It was reported that cytolytic/cytotoxic effects of snake components on whole cells are connected with the consequences of oxidative stress. This can be manifested by the modulation of signaling pathways that are linked to cell viability [22,23], overproduction of reactive oxygen species [24,25], and mitochondrial function damage [10,26,27].

The components of snake venom offer great therapeutic potential, but at the same time, they carry the risk of causing unfavorable reactions inside an organism; in particular, they can trigger an immune reaction. Therefore, it raises the need to define precise mechanisms of action of venom proteins on human cells, identified individually for each group of components. 

The aim of the study was to determine the effect of the three-finger toxins from *Naja ashei* venom on the human cells of the immune system-monocytes (U-937) and promyelocytes (HL-60). The particular emphasis of the experiments was to concentrate on the modification of cell membranes under the influence of various doses of this toxin. Based on the previous studies suggesting that 3FTxs may damage or modify the membrane structure, changes in the physicochemical parameters that characterize the membranes under three-finger toxin proteins treatment were investigated using model membranes (that mimic the U-937 and HL-60 native membranes). Next, the real level of membrane damage was examined by in vitro tests (through MDA concentrations and LDH activity). The obtained results describing the state of membranes were compared with biochemical indicators, leading to the determination of the total physiological effect of 3FTxs on tested human cells (cell survival, induction of inflammation, medial lethal dose LD50).

## 2. Results

### 2.1. Fractionation of Crude Naja ashei Venom and Identification of Proteins in the Obtained Fractions

**Figure 1:**
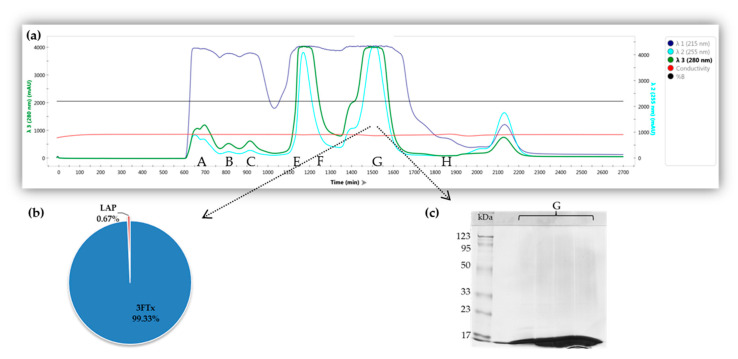
Crude venom size exclusion chromatography (SEC) separation and protein identification schemes in Fraction G: (**a**) SEC chromatogram obtained for the separation of *Naja ashei* venom using Sephadex G75 Superfine. The fractions collected during the experiment were marked by labels (A–H) below the peaks; (**b**) Pie chart presents the results of the quantitative MS analysis for the G fraction; 3FTx (three-finger toxins); LAP (low abundant proteins)—proteins whose total percentage share did not exceed 1% were included in the LAP group. The complete list of proteins with its quantitative data is provided in the Supplementary Materials; (**c**) SDS-PAGE of the collected chromatographic samples belonging to Fraction G.

Size-Exclusion Chromatography (SEC) was used to reduce the complexity of *Naja ashei* venom and obtain samples with a high proportion of three-finger toxins. Protein separation was monitored at 215, 255, and 280 nm. However, as the former wavelength signal was almost constantly saturated, the two latter ones were used to differentiate the peaks on the chromatogram (Figure 1a). Then, to reveal the homogeneity level in collected samples, a protein band pattern analysis was performed via an SDS-PAGE experiment. Chromatographic separation yielded seven fractions in total, namely A–H (initially, the D fraction was also collected, but due to the very low protein concentration it was rejected). Protein composition in the obtained fractions was determined by shotgun LC-MS/MS analysis. MS data analysis demonstrated that the G fraction exhibited the highest proportion of proteins from the three-finger toxin family. Overall, the Andromeda search engine identified peptides that can be distributed among 20 different proteins, almost half of which (9 proteins; 45%) are members of the 3FTx family. Nevertheless, quantitative analysis (precursor intensity-based) indicated that three-finger toxins constitute 99.33% of all proteins in the fraction G (Figure 1b). Those results are in agreement with the position of protein bands on the obtained gel images, where all visible proteins were located in the lowest region of the gel (Figure 1c). Therefore, fraction G was selected for further functional analyses.

### 2.2. Characterization of Model Membranes and Their Physio-Chemical Parameters

#### 2.2.1. 3FTx Interaction with Multicomponent Monolayers

In order to understand how 3FTx proteins interact with membranes, HL-60 and U-937 model membranes were created at the air-water interface. Lipids were compressed to obtain surface pressure isotherms. A mixture consisting of pure phospholipids, reflecting the composition of the natural membranes HL-60 and U-937, was adopted as the control system. The shapes of these isotherms are typical for the studied models and can be found in the literature [28]. Previous studies performed on the HL-60 and U-937 cell lines suggested a higher sensitivity, identified for HL-60 line, in contrast to the U-937 line.

**Figure 2:**
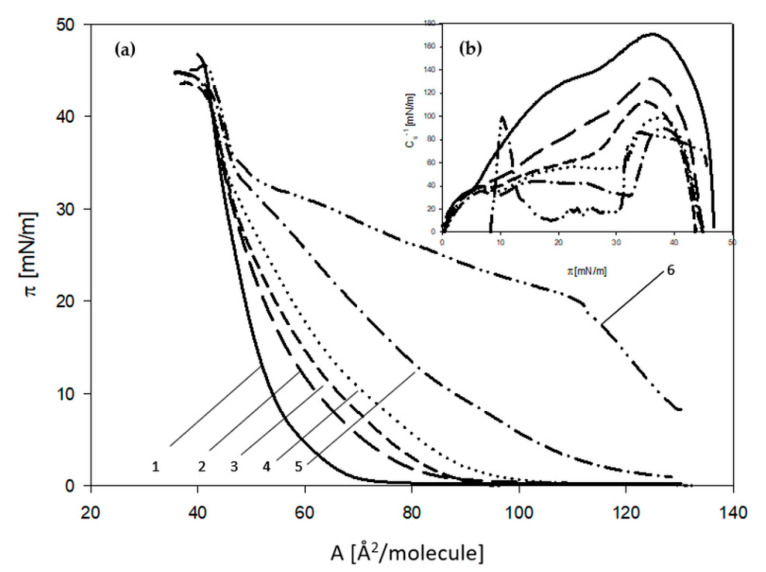
(**a**) Surface pressure isotherms (π) as a function of the area per lipid molecule (A) and (**b**) the dependencies of the static compression modulus (Cs^−1^) vs. the surface pressure (π) obtained for HL-60 cell line. Phospholipid monolayers spread on phosphate buffer—(1) solid line and on phosphate buffer with 5 ng/mL 3FTx; (2) long-dashed line, 10 ng/mL 3FTx; (3) short-dashed line, 20 ng/mL 3FTx; (4) dotted line, 40 ng/mL 3FTx; (5) dotted-dashed line, 100 ng/mL 3FTx; (6) double dotted-dashed line.

For this reason, the analysis of physicochemical parameters was started for the HL-60 model membrane. First, the changes in membrane properties depending on the different 3FTx concentration were measured (Figure 2).

**Figure 3:**
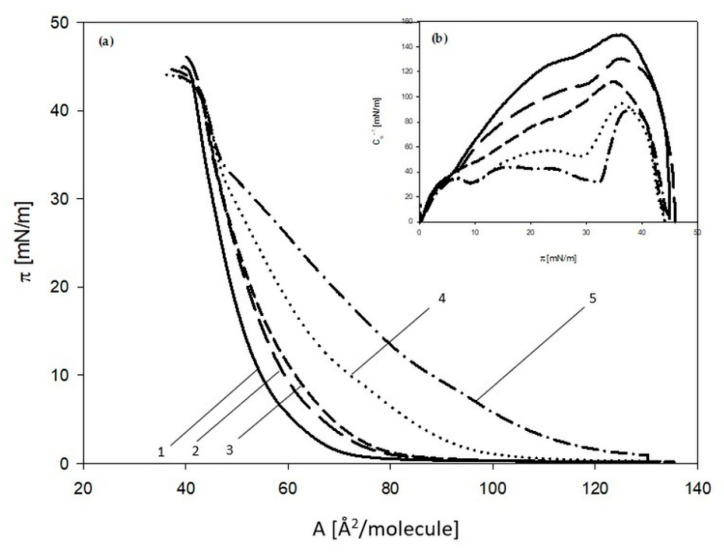
(a) Surface pressure isotherms (π) as a function of the area per lipid molecule (A) and (b) the dependencies of static compression modulus (Cs^−1^) vs. surface pressure (π) obtained for U-937 line. Phospholipid monolayers spread on phosphate buffer—(1) solid line and on phosphate buffer with 5 ng/mL 3FTx; (2) long-dashed line, 10 ng/mL 3FTx; (3) short-dashed line, 20 ng/mL 3FTx; (4) dotted line, 40 ng/mL 3FTx; (5) dotted-dashed line.

Changes in the isotherm shape (compared to the control system) were noticed when lipid monolayers were spread on subphase with 5 ng/mL of 3FTx fraction. This concentration was taken as the minimum. Above this concentration, the curves obtained for monolayers were shifted towards larger areas per lipid molecule. There were also changes observed in the LC (liquid crystal) state of the membrane when the distance between lipids was very small. Increasing concentration of 3FTxs caused a higher degree of packing of lipid components at the lower surface pressures. A different shape of the isotherm was demonstrated for 3FTxs at the 100 ng/mL, which is close to the designated medial lethal dose. The applied concentration raised the surface pressure value above zero, already in the gaseous state of the monolayer. This increase in surface pressure, even in relatively large molecular regions, may indicate the maintenance of aggregation of molecules that create the monolayer or high surface activity of peptides [29]. For this reason, further analyses, performed analogously for the model membrane U-937 (Figure 3), do not include the concentration of the toxin close to the medial lethal dose.

The shape of isotherms obtained for U-937 has changed and depends on the increasing concentration of the 3FTxs, similar to that shown for the HL-60 model. On the basis of the π-Å isotherms, the physicochemical parameters characterizing the membrane were determined.

In Table 1, the values of the area per lipid molecule in the membrane at the moment of its maximum compression are presented. The percentage of change in the second parameter—A_30_, obtained at surface pressure, corresponding to the values of native biological membranes, 30 mN/m—is shown on Figure S2a. In the range of 30–35 mN/m, the monolayer can form a compact, homogeneous structure, which is sufficiently stiff to demonstrate the changes of membrane structural parameters under the 3FTx treatment. The value of the static compression modulus (Cs^−1^), determined at the reported pressure, proves that the condensation of the monolayer is similar to the native membrane density.

Thus, it can be assumed that the lipid monolayers, obtained by the Langmuir technique, mimic the outer surface of the native membrane of the cell [30].

From the presented results for parameters A_lim_ and A_30_, statistically significant changes in the parameter were observed only for a concentration of 10 ng/mL (compared to the control). Then, in the case of isotherms for HL-60 and U-937, the change was almost the same, differing only by about 4%. The greatest differences were noted at 40 ng/mL of 3FTx fraction. For the HL-60 line, a slightly greater increase in the A_30_ parameter (by 18%) was observed, while for the second analyzed model membrane, it was an increase of 15%. It has been shown that the greater the concentration applied fraction administered, the more the A_lim_ and A_30_ parameters values increase.

The second of the analyzed parameters was the surface pressure, at which the monolayer collapses—π_coll_. The recorded values of the presented parameter slightly differentiated both tested models. These slight changes in the range of approx. 0.3–2 mN/m are statistically significant.

The results of the Cs^−1^ parameter are also presented in Table 1. Similarly to the A_30_ parameter, the static compression modulus was calculated for membranes at the state of π = 30 mN/m (Figure S2b).

The results obtained for both the HL-60 and U-937 models indicate a concentration-dependent reduction of the Cs^−1^ value by 3FTxs. Similar but statistically significant changes in the parameter were noted. For example, the greatest reduction was associated with the concentration of 40 ng/mL 3FTxs-by 75.4% for U-937 and by 77.5% for HL-60 in comparison to the control.

#### 2.2.2. 3FTx Interaction with Single-Component Monolayers

The main purpose was to determine which membrane lipid component is most important in 3FTx-membrane interaction. In order to thoroughly understand the investigated mechanism described for studied cells, single-component monolayers were prepared. 

**Figure 4:**
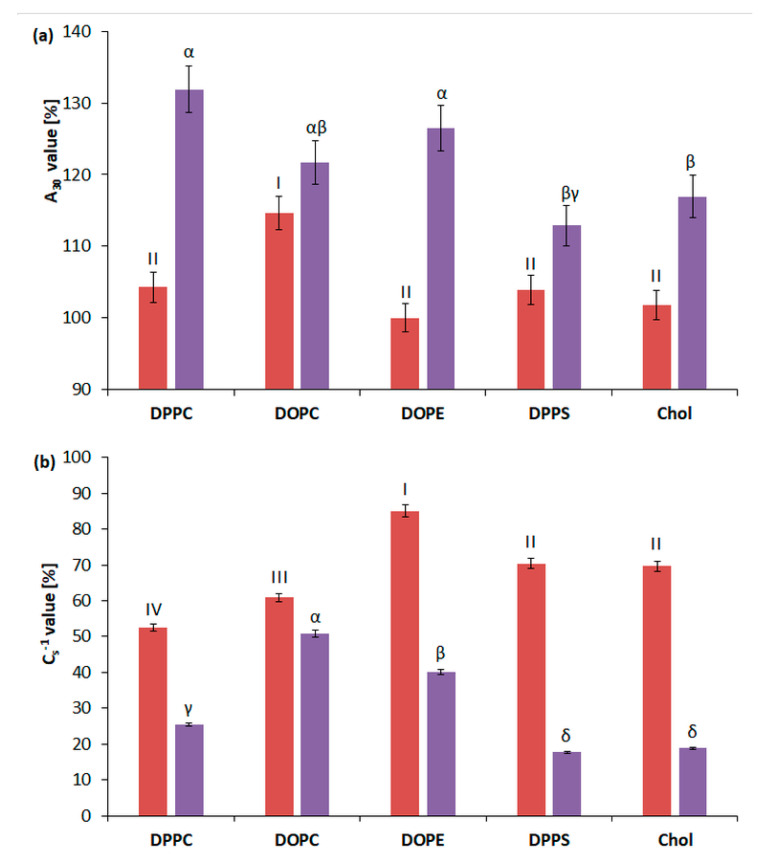
Effect of 3FTxs (at a concentration of 10 ng/mL—red color; at a concentration of 40 ng/mL—purple color) (**a**) onthe limiting area (measured at for π = 30) and (**b**) on static compression modulus vs. surface pressure (C_s_^−1^) for representative lipids (phosphatidylcholine (DPPC, (16:0)), phosphatidylcholine (DOPC, (18:1)), phosphatidylserine (DPPS), phosphatidylethanolamine (DOPE), and cholesterol (Chol). Values represent the average ± SD (n = 5). Statistically significant differences between tested lipids were marked: for the 10 ng/mL 3FTx concentration, Roman numerals were used; for the 40 ng/mL 3FTx concentration, the letters of the Greek alphabet were used.

To determine the interaction between 3FTx and membrane components, phospholipids with different composition of hydrophilic (polar) parts (PE and PS (Figure S3a) and PC (Figure S3b)) and with various hydrophobic parts (containing 16:0 saturated and 18:1 unsaturated fatty acids (Figure S3b)) were chosen. Particular attention was paid to the interaction with choline lipids, because in the membranes of the tested HL-60 and U-937 cell lines their content is the highest. Due to the different percentages of cholesterol in the membranes of the tested cells, the effect of 3FTxs on this lipid was also checked (Figure S3c). On the basis of the previously presented results for multi-component systems, two concentrations were selected for further analysis: 10 ng/mL and 40 ng/mL, at which the most interesting changes in physicochemical parameters were observed. The parameters A_30_ and Cs^−1^ calculated for the single-component membranes are presented in the Figure 4a,b.

For the applied concentrations of 3FTxs, an increase in A_30_ with a simultaneous decrease in the value of Cs^−1^ was noted.

For 10 ng/mL of 3FTxs, parameter A_30_ increased by about 4% for almost all types of tested lipids compared to the control (lipid spread on subphase without 3FTxs). The DOPC lipid was an exception, for which the increase in the area per molecule was higher (14.6%). The concentration of 40 ng/mL caused more intensive interaction with the tested choline lipids. It was indicated by a significant increase in the A_30_ parameter and greater reduction (more than for 10 ng/mL 3FTx) in the values of the Cs^−1^ parameters for the tested lipids.

Furthermore, the greatest interaction was demonstrated for zwitterionic lipids containing choline, DPPC (by approx. 31%), as well as DOPE (26.47%), containing ethanolamine in the polar part. Similarly, for the second tested choline lipid-DOPC, an increase of approx. 21% was reported. The increase in A_30_ value was also noted for Chol (16.9%) and for the negative lipid—DPPS (12.8%). According to Cs^−1^, the greatest change that was observed in relation to control was 82% for DPPC and Chol, respectively, and the smallest for DOPC lipid (60.1%).

#### 2.2.3. Calculation of Gibbs Excess Free Energy of Mixing

**Figure 5:**
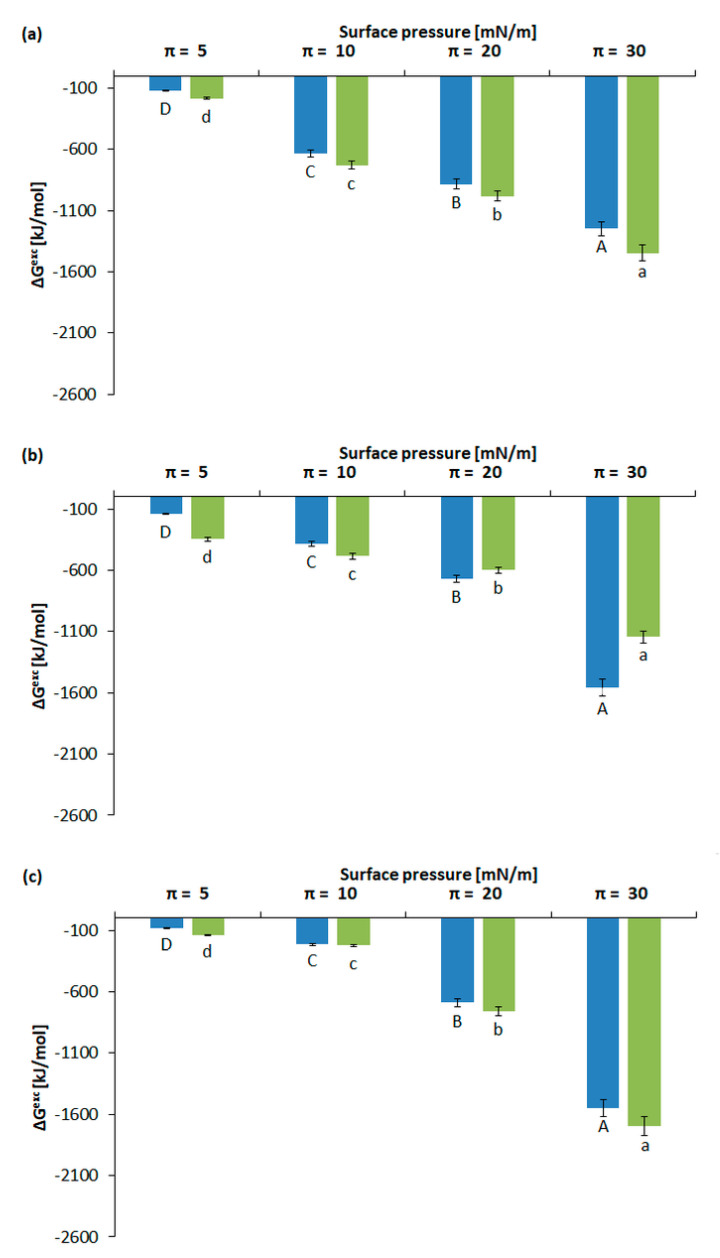
The changes of excess free energy of mixing (ΔG^exc^) for the HL-60 model membrane (green color) and U-937 model membrane (blue color) (**a**) without 3FTx treatment and with (**b**) 10 ng/mL 3FTx and (**c**) 40 ng/mL 3FTx at four surface pressures: 5, 10, 20, and 30 mN/m. Values represent the average ± SD (n = 5).Significant differences between surface pressures were marked: within the U-937 cell line they were marked by uppercase letters and for the HL-60 cell line with lowercase letters.

To describe the thermodynamic effects associated with the presence of 3FTxs in contact with membranes, the excess free energy of mixing was calculated for model monolayers of the HL-60 and U-937 cell lines (Figure 5a–c). For all mixed monolayers, negative values of ΔG^exc^ were noted. The most negative value was observed at π = 30 mN/m and statistically significant changes were found between the tested models. For the concentration of 10 ng/mL, the values for U-937 were more negative than for HL-60, i.e., approx. (−1557 kJ/mol). Although for the concentration of 40 ng/mL, the values for both tested models were still negative and, interestingly, for the HL-60 line, in this case, they reached a more negative value (–1694.94 kJ/mol).

### 2.3. Biochemical Characterization of Membrane Properties Described by Lipid Peroxidation (MDA Concentration) and Membrane Damage (Lactate Dehydrogenase (LDH) Assay)

**Figure 6:**
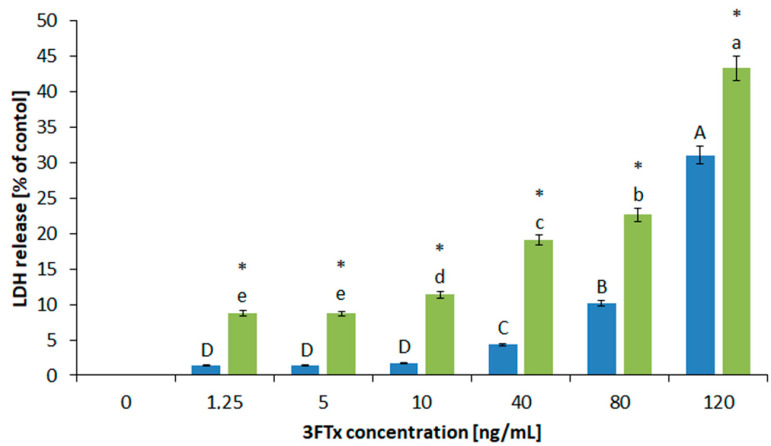
Concentration-dependent LDH release from HL-60 (green color) and U-937 (blue color) cells treated by 3FTx in six different concentrations. Values represent the average ± SD (n = 5). Different letters indicate significant differences between 3FTx concentrations: for the U-937cell line, uppercase letters; for the HL-60 cell line, lowercase letters. Asterisks indicate a significant difference between the tested cell lines (*p* ≤ 0.05).

Changes in parameters that characterized the native membranes of the tested cell lines under the 3FTx treatment were determined by the level of membrane damage (the LDH test), as well as by the concentration of dimalonaldehyde (MDA)—an indicator of membrane lipid peroxidation. Membrane disruption as a result of cell damage is assessed by the leakage of the cytoplasmic enzyme LDH (Figure 6). 

The data confirmed the strong, destructive effect of 3FTxs on both cell types. For all tested concentrations of 3FTxs, the amount of released LDH was significantly greater for the HL-60 line (than for the U-937 line). The obtained LDH values were closely correlated with the increase in the dose of 3FTxs. It was observed that the application of the smallest doses at the 1.25 ng/mL of protein fraction, significantly damaged cells, especially the HL-60 cells. For the U-937 line, the activity of the released LDH enzyme in the concentration range of 1.25–10 ng/mL of 3FTxs is not changed. Further membrane-related processes of damage are observed from the dose of 40 ng/mL 3FTx. 

**Figure 7:**
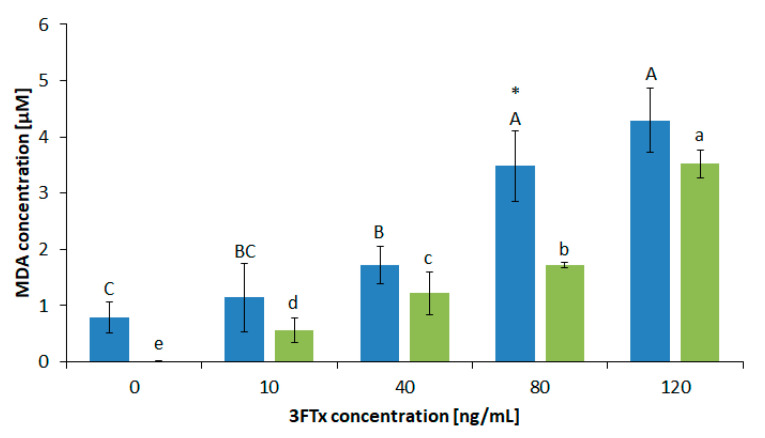
The extent of peroxidation of membrane lipids expressed by MDA content in HL-60 (green color) and U-937 (blue color) cells after treatment with 3FTx in six different concentrations. Values represent the average ± SD (n = 5). Different letters indicate significant differences between 3FTx concentrations: for U-937cell line, uppercase letters; for HL-60 cell line, lowercase letters. Asterisks indicate a significant difference between the tested cell lines (*p* ≤ 0.05).

The opposite relationship for both cell lines is illustrated on the graphs showing the results of the MDA test. The parameter was not determined for lower concentrations of 3FTxs, because the activity of LDH has clearly changed its values from 10 ng/mL (in both cell lines). Therefore, this concentration was considered significant for further measurements that characterize the biochemical properties of cell membranes. The LDH test indicates that higher values of this parameter were present for the HL-60 line, but the level of peroxidation of membrane lipids of these cell lines was significantly lower (in comparison to the U-937 line). As was shown in Figure 7, for the HL-60 cells treated with 10 ng/mL of 3FTxs, MDA production was higher by 0.51µM compared to the control. 

At the same concentration, the presented amount of MDA for U-937 increased by 0.35 µM in comparison to 3FTx untreated cells. It is worth noting that a dose of the toxin above 80 ng/mL did not cause statistically significant differences in the production of MDA in U-937. However, the increasing concentration of 3FTxs, close to the LD50 dose, is important for HL-60 cells and their degree of further membrane damage.

### 2.4. Human Cell Culture Tests under 3FTx Treatment 

#### 2.4.1. Determination of the Cell Viability (XTT Test) 

**Figure 8:**
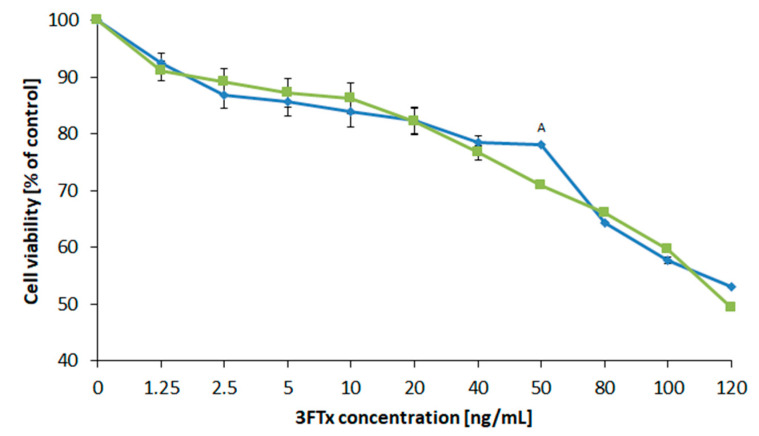
Viability of HL-60 (green color) and U-937 (blue color) cells. Values represent the average ±SD (n = 5). The letter A indicates a significant difference between the tested cell lines (*p* ≤ 0.05).

The cytotoxic effects of the 3FTx fraction were measured by exposing the human cell lines U-937 and HL-60 to 10 different concentrations of *N. ashei* three-finger toxins (1.5–120 ng/mL). Concentrations for this test were selected in accordance with the doses proposed by Das et al. [21,31]. The test results presented as the percentage of viable cells in relation to the control (Figure 8) showed that the survival of both tested cell lines decreased with an increasing concentration of the 3FTxs. The decrease in viability by approx. 9% was recorded at the lowest concentration, which was 1.5 ng/mL. From 5 to 20 ng/mL of 3FTxs, the decrease in cell viability compared to untreated cells was almost 15–18%. For all tested cells, the highest applied concentration of 3FTxs–120 ng/mL caused a decrease in the viability by 50%.

#### 2.4.2. Calculation of the Medial Lethal Dose (LD50)

The viability, as determined by the XTT method, correlates with the number of survived cells. The observed results for the highest concentration used in the measurements (120 ng/mL) allow for the assessment of the toxicity of the tested protein fraction in biological systems. Based on the presented results, the median lethal dose (LD50) was determined using the Behrens method [32], which is presented in Table 2. 

The amount of the chemical substance (statistically calculated on the basis of the test results) that cause death in 50% of organisms after a single dose treatment for U-937 cells is 4.5% higher than for the second tested cells. This indicates that HL-60 cells are more sensitive to 3FTxs. Another parameter, the concentration of NO_x_, which indicates the induction of inflammation, may be an additional measure of the toxic effects caused by 3FTx on cells. The nitric oxide production was analyzed in selected concentrations (10, 40, 80, and 120 ng/mL) and is presented Figure S4. Both human cell lines have the ability to produce NO_x_ in similar concentrations. Increasing the dose of 3FTxs into systems generated more intensive inflammation.

## 3. Discussion

According to the pharmaceutical potential of animal venoms, it can be concluded that the complex mixture of venom proteins could be an important source of therapeutic substances. Undeniably, the anti-tumor activity of snake venom and its cytotoxic components, such as 3FTxs, is one of its most attractive properties, which draws scientific attention and, as a result, accelerates the development of new therapeutic strategies. However, a particularly important element in the development of safer medicinal substances based on venoms is to understand how those proteins influence cell membranes. 

Due to the interaction between 3FTxs and the lipid bilayer, disruption of membrane integrity may consequently affect a physiological change in overall cell metabolism. The complex composition of the lipids in the cell membrane (different phospholipid structure and cholesterol content), and very dynamic bilayer properties create the possibility of conformational changes of proteins. For these reasons, lipid–lipid and lipid–protein interactions are difficult to investigate [33,34,35]. Taking into account the above aspects, not only experimental approaches (biochemical indicators) were applied, but mainly physicochemical modeling (Langmuir monolayers). The most common lipid components of animal cell membranes were selected to reflect the effect of 3FTxs on the individual membrane components. Determination of their possible interactions was investigated in several measurements, including not only the 3FTx, but also phospholipid chemical structure. The lipids have been selected precisely to study the mechanism of 3FTx influence on membrane depending on the presence of i) different polar groups (PC, PE, and PS) and ii) different hydrophobic parts (PC 16:0 and 18:1). 

From a concentration of 5 ng/mL, 3FTx interaction with the membrane lipid components can be observed. Native animal cell membranes exist naturally in a liquid crystal state [36,37], and changes in the determined physicochemical parameters of the membrane at the naturally occurring pressure values (30 mN/m) clearly indicate the influence of 3FTxs on the membrane surface. The phospholipid components are better organized and the distances between them have progressively reduced. 

As a consequence, the expanded liquid state of the one-component monolayers was achieved at lower surface pressures, in the order PS < PE ~ PC. Konshina et al. [15,38] hypothesized that the mosaic of the membrane surface (mainly due to the presence of one or more types of anionic lipids) may contribute to the adaptation of two amphiphilic systems-cytotoxins and membrane lipid components. Similarly, a negative-positive charged polar part presented by zwitterionic lipids in membranes is one of the important determinants of toxin binding to the membrane. 

The given test results correlate positively with indicated favorable interaction of 3FTx with the lipid polar parts in the literature [38,39,40]. The results confirmed that the choline phospholipids, the main component of animal cell membranes, are the most important in the mechanism of action of 3FTxs on membranes, especially the zwitterionic type. The results also indicate the interaction between 3FTxs and another lipid with a zwitterionic charged DOPE. In the case of phospholipid with a negative polar group, namely DPPS, this interaction was slightly weaker. Analyzing the modification of the structure of the model monolayers, the effect caused by 3FTxs is intensively observed with the increasing concentration of the tested proteins.

Interestingly, at the lower concentrations of the toxins (approx. 10 ng/mL), not the polar, but the hydrophobic part of the membrane plays a dominant role in the interaction with 3FTx. This dependency has been shown in the different results obtained for the structural parameters of monolayers composed only from lipids with saturated or unsaturated fatty acids (PC 16:0 and 18:1). These effects can be masked by the influence of the polar group of lipids if higher concentrations of 3FTx are used (close to approx. 40 ng/mL). It can be concluded that the degree of the introduced surface-structural changes is more dependent on the charge of the lipids polar parts.

The results show that the phospholipid:cholesterol ratio in the membrane may play an important role in the damage of the cell membrane structure caused by snake venom and/or lead to cytotoxicity [41,42,43]. It is worth adding that the physicochemical parameter changes obtained for cholesterol-3FTx systems were very similar to the results discussed for other single-component monolayers. The presence of Chol contributed to the organized character of the membrane and, under 3FTx treatment, significantly contributing to its stiffening. It appears that the differential affinity of 3FTxs for the individual membrane components is important, especially in the context of specific membrane functions. On the other hand, the different lipid compositions of the natural membranes may initiate different 3FTx activity on the tested cell types.

Based on the Monte Carlo simulations method, Dubovskii et al. [17] showed that the specific distribution of hydrophobic and/or hydrophilic residues in cytotoxins may promote their ability to localize inside lipids bilayer. It should be emphasized that the distribution of non-polar residues, e.g., in cytotoxins, which favor deposition on membranes, depends not only on the amino acid composition of the studied protein, but also depends on the spatial configuration and the arrangement of the 3FTx loops (especially loops I and II). The typical shape of loop II, present in all experimentally derived three-dimensional cytotoxin structures, has the ability to interact with membranes and plays a key role in the mechanism of penetration into bilayer systems [44]. Referring to the lipid membrane compositions from each of the studied cell lines, simplified systems of artificial membranes (monolayers) have also been prepared to mimic the natural lipid membrane. 

In relation to the U-937 and HL-60 lines, the obtained results clearly prove that the tested 3FTx fraction interacts with a mixture of different lipids, mimicking their membrane. In the case of the tested membrane models, the degree of lipid packing in the monolayer changed and the surface pressure increased, which, in the case of proteins, is a direct proof of protein–monolayer interaction.

The intensity of the interaction of 3FTx with the components of monolayers increases with the concentration of the tested protein fraction in the hydrophobic environment of the membrane (similar to what was demonstrated for the one-component systems). Modification of the structure of model systems gives the basis for the conclusion that the studied protein fraction has a different influence on both tested membranes, especially their stiffness and flexibility. Such dependence may suggest a different form of native cell membrane adaptation in presence of 3FTx, and thus, a physiological response differentiating both cell lines. It was shown that the model U-937 cell membrane was slightly more compressed compared to the HL-60 line. The direct cause influencing membrane behavior may be the content of individual lipid components. The membrane of the HL-60 line has more phospholipids with unsaturated fatty acid and a high percentage of cholesterol in comparison to the U-937 native membrane. In the study by Lee et al. [45], it was suggested that some cardiotoxins were identified as cholesterol-sensitive. It means that an increase in the level of cholesterol in the membrane promotes and significantly improves endocytosis in the cell. This phenomenon has been determined to be evolutionarily beneficial for those toxins that can affect different cell types or intracellular membrane organelles. Likewise, lipid rafts, which are cholesterol-rich microenvironments of the cell membrane, have been identified as areas playing a role in this internalization process [46,47]. Due to the favorable interaction of 3FTx with cholesterol as well as with unsaturated fatty acid lipids, as has been shown, structural effects described for the HL-60 model membrane likely may suggest a more toxic effect and a greater sensitivity to 3FTxs.

The electrostatic effects associated with the 3FTx proteins depend on the presence of zwitterionic choline lipids (54.4% U-937 and 48% HL-60). Due to the lower percentage of anionic lipids (DPPS) in both tested membranes (12.8% U-937 and 15% HL-60), compared to other lipids, positively charged proteins part more often and effectively interact with the anion part presented in zwitterionic lipids.

The obtained negative values of Gibbs excess free energy of mixing confirm the hypothesis concerning the possibility of both-electrostatic and non-polar interaction of HL-60 and U-937 membranes with the tested protein family. In the determined parameter, positive values indicate an unfavorable interaction between mixed components, while negative values indicate a more favorable one. It is reported that favorable interactions between the 3FTxs and the membrane lipids were obtained in both tested models. At lower fraction concentrations, more favorable interactions are obtained for the U-937 model, but for higher concentrations, more favorable interactions were reported for HL-60.

The demonstrated differential interaction of model membranes with 3FTxs was verified by establishing biochemical and physiological effects in native cells. If the venom components will be used for medical purposes, it is necessary to define its action in natural systems, not just in the model membrane. Biochemical parameters need to be provided to inform about the consequences caused by 3FTx.

For this reason, the influence of 3FTxs on the native membranes was described by indicators characterizing the native membranes, such as (i) MDA, which informs about the peroxidation state of the cell membrane, and (ii) LD, the presence of which in the cell supernatant informs about the membrane disintegrity. Interestingly, the degree of lipid peroxidation obtained for U-937 cells was higher than the values of these parameters obtained for HL-60 cells. It is caused by the presence of the higher content (expressed as mol%) of membrane phospholipids with multiple bonds in fatty acid (i.e., lipids 20:4 and 18:2 in U-937 cells than in HL-60 [48,49]. Greater exposure to multiple bonds and their frequency in hydrophobic parts of lipids reduces the probability of their damage by peroxidation, and thus, increases the concentration of the lipid peroxidation product: dimalonaldehyde.

However, higher LDH values were noted for the HL-60 cells, so their membrane was more damaged. X-ray analyses by Bilwes et al. [50] and studies by Konshina et al. [15] and Lee et al. [45] showed that the CTx structure is formed by trimers with a hydrophobic outer surface and hydrophilic pores on the inside. The spatial arrangement of positively charged parts of CTx proteins (or their hydrophobic domains) makes it possible to interact with the membrane. This gave the hypothesis that the various CTx effects obtained from cobra venoms could be realized through the formation of pores or channels in membranes by the interaction with phospholipids. Membrane pores formed by CTx may have a limited lifetime on the cell surface due to membrane reorganization [51]. This mode of protein–membrane interaction, resulting from favorable interactions between components (as evidenced by the Gibbs negative free energies discussed earlier), may result in a greater outflow of LDH to the extracellular environment for the HL-60 cells. Moreover, cytotoxins isolated from the venoms of other Elapidae snakes: *N. oxiana*, *N. kaouthia*, and *N. haje* have been shown to easily penetrate into living cancer cells (i.e., A549 human lung adenocarcinoma and promyelocytic leukemia HL-60; diagnosed by confocal spectral imaging) and accumulate significantly in their lysosomes [52,53]. The demonstrated ability to accumulate venom toxins may suggest consequences of their presence, e.g., cytotoxic effects. Previous studies have shown that human myeloid leukemia cells are highly susceptible to the cytotoxicity of cytotoxins [54,55,56]. Moreover, it has been shown that the cytotoxicity of certain venom components—also belonging to the 3FTx family—induces apoptosis in neoplastic cells via a pathway mediated by mitochondria, lysosomes, or damage to the cell membrane [27,54,57]. The obtained results for the U-937 and HL-60 lines confirmed the significant cytotoxic effect of 3FTxs, which had previously been suggested in studies on HEK 293 and L6 cells [21]. The administration of a dose of 100 ng/mL over 24 h resulted in a reduction of the viability of cells, especially the L6 line, to the level of 85%. HL-60 cells are most sensitive to 3FTxs, while U-937 cells show a slightly higher survival (indicated by a dose of 50 µg/mL, which differentiates the two cell lines). The use of the minimum dose of 1.25 ng/mL resulted in decreased survival in both lines within 24 h. The confirmation of the induction of the cytotoxic effect is the high concentration of NOx recorded for both lines. This effect was previously noted for some elapid venoms [58,59]. This indicates that the 3FTx fraction induces inflammation; however, the production of NOx in response to the action of 3FTxs is not the major differentiating factor for the studied cell types. The immunomodulatory effect of the 3FTx fraction requires further research. A final measurement related to the determination of the effect of 3FTx on cell lines was to determine the safety of administration of venom components by determining the LD50. Studies conducted by Okumu et al. [60] have shown a dose-dependent and time-dependent variable toxicity of the components of the *Naja ashei* venom. The LC50 for this snake venom was 63.02 μg/mL. According to the Clarkson Toxicity Index [61] and the Meyer Index [62], the venom was classified as highly toxic. Tests performed with the human cell line U-937 showed that the LD50 of the cells was 126.8 ± 2.294 µmol/L/1 × 10^6^ 3FTx. Higher sensitivity was detected for the second tested cell line—HL-60. The obtained LD50 value was lower and amounted to 121.29 ± 1.42μmol/L/1 × 10^6^ 3FTx. According to the parameter definition, half the number of cells has been damaged, and above this dose, cells may also be destroyed. The obtained data clearly confirm that it is the membranes of the HL-60 cells that are more sensitive to 3FTx and that this type of cells is more susceptible to the cytotoxic effect of 3FTx.

Using various characterization methods, the studies were carried out in model systems on in vitro cell lines (U-937, HL-60) and Langmuir monolayers which refer to the membranes of these cells. The assessment of the membrane–3FTx interaction may help other scientists better understand the first step of the mechanism of toxicity and may aid in developing therapeutics, i.e., antivenoms. Since the applied systems are significantly simplified models, they have some limitations. First of all, the whole cellular environment found in organisms is not taken into account. In the case of cell lines, the influence of other cells (e.g., intercellular junction, paracrine, and hormone signaling) is disregard. According to Langmuir monolayers, the direct membrane surrounding environment is not taken into account, and the composition of the membrane is also simplified.

On the other hand, the simplification of research systems allows for precise observation of the specific mechanisms or stages of mechanisms, without disruptions, precisely. They result from the other processes that can add up or mask certain effects in model systems. They allow for the interpretation of the extent to which interaction between tested factors and cell elements occurs, under controlled conditions (while eliminating the influence of other environmental factors).

## 4. Materials and Methods

### 4.1. Venom Collection

The venom sample was acquired from two adult *Naja ashei* specimens (male and female) captured in Kenya. Venoms were extracted three times from each individual (mean volume: 2.25 mL) and then pooled. Snakes were kept in the breeding garden Pata near Hlohovec (Slovakia), which had been designed for reptiles′ conservation of the gene pool under the veterinary certificate No. CHEZ-TT-01. The breeding garden also serves as a quarantine station for imported animals and is an official importer of exotic animals from around the world, having the permission of the State Nature Protection of the Slovak Republic under the No. 03418/06 to trade with endangered species of wild fauna and flora and on amendments to certain laws under Law no. 237/2002 Z.z. The samples were frozen to −20 °C immediately after milking (transport temperature) and then stored at −80 °C until use.

### 4.2. Fractionation of Naja ashei Venom with Size-Exclusion Chromatography

The amount corresponding to 100 mg of venom proteins was diluted to a final volume of 3 mL with phosphate-buffered saline (PBS; 0.1 M NaCl, 50 mM sodium phosphate buffer, pH 7.0). Size-exclusion chromatography was performed on a 1.6 × 100 cm glass column filled with Sephadex G75 Superfine bed using NGC Chromatography System (Bio-Rad, Hercules, CA, USA). A constant flow rate of PBS at 0.1 mL/min was applied to maintain protein elution. During the process, fractions of 2 mL were manually collected. After chromatographic separation, the composition of each sample was monitored using SDS-PAGE on 13% resolving gels (with 5% stacking gels) according to the standard procedure. Fractions that shared the same protein band patterns and originated from the same peak were combined and concentrated on the centrifuge filters Vivaspin 2 with membrane 3000 MWCO PES (Sartorius Stedim Lab Ltd., Stonehouse, UK). 

### 4.3. Identification of Proteins in Obtained Fractions Using Shotgun LC-MS/MS Analysis

Prior to digestion, proteins in fractions were precipitated with six volumes of acetone for 1 h at –20 °C, and then reconstituted in 100 mM ammonium bicarbonate buffer, pH 8. Protein concentration in each fraction was measured with Pierce™ BCA Protein Assay Kit (Thermo Scientific, Waltham, MA, USA) according to the manufacturer’s instruction. A detailed description of the procedure of the LC-MS/MS shotgun experiment with data analysis can be found in [8]. Briefly, for trypsin proteolysis, each sample contained 4.3 μg of proteins in 25 uL of 100 mM ammonium bicarbonate pH 8. Protein reduction and alkylation were carried out with DTT and IAA, respectively. Then, 0.86 μg of digested peptides from each sample was separated on a Dionex Ultimate 3000 RSLC NanoLC system (Thermo Fisher Scientific, Waltham, MA, USA) using an Acclaim PepMap RSLC nanoViper C18 column (75 μm × 25 cm; 2 μm) (Thermo Fisher Scientific, Waltham, MA, USA) and detected on a Q Exactive Orbitrap mass spectrometer (Thermo Scientific, Waltham, MA, USA) in data-dependent acquisition (DDA) mode.

MaxQuant software (ver. 1.6.7.0) was used for qualitative and quantitative analysis of the raw MS/MS files of each fraction. Acquired peak lists were search against UniProtKB Serpentes database (release 9/2019). Peptide quantification relied on iBAQ (intensity-based absolute quantification) values. Razor and unique peptides were used to estimate the amount of particular protein in the sample. iBAQ values of identified proteins belonging to the same protein group was summed and divided by iBAQ values of all identified proteins, thus determining the percentage of specific protein groups in fractions.

The mass spectrometry proteomics data have been deposited to the ProteomeXchange Consortium via the PRIDE partner repository with the dataset identifier PXD023801 and 10.6019/PXD023801. The uploaded dataset contains proteomic information concerning all collected fractions (A–C, E–H).

### 4.4. Model Membranes Composition

Based on the data published in the literature concerning the lipid profiles of two human cell lines (for HL-60 [49,63,64] and for U-937 [65,66]), multi-component systems imitating membranes of the above-mentioned cells were prepared. High purity, synthetic lipids were chosen for studying the impact of 3FTx on model lipid systems: 1,2-dipalmitoyl-sn-glycero-3-phosphocholine 16:0 (DPPC), 1,2-dioleoyl-sn-glycero-3-phosphocholine 18:1 (DOPC), 1,2-dioleoyl-sn-glycero-3-phosphoethanolamine 18:1 (DOPE), 1,2-dipalmitoyl-sn-glycero-3-phospho-L-serine 16:0 (DPPS), and Cholesterol (Chol) were purchased from Avanti Polar Lipids. The solvents (chloroform, methanol (POCH (Avantor Poland)) of chemical purity were used to prepare PC, PE, and Chol lipid solutions and were dissolved in chloroform, while PS were prepared in a 9:1 v/v chloroform:methanol solution. The phosphate buffer (PBS; 0.1 M, pH = 7.0) were used as a subphases. The isotherms depending on the 3FTx concentration (5, 10, 20, 40, and 100 ng/mL) were obtained for HL-60 and U-937 mimicking model systems. Based on preliminary results, isotherms for single lipid components were obtained for 10 and 40 ng/mL 3FTx. The cholesterol and phospholipid (PL) ratio for the membrane mimicking system of used human cell lines were 0.35 for HL-60 and 0.29 for U-937. Mixtures were prepared from the respective stock solutions as follows: HL-60: 35%mol Chol, 65%mol phospholipids (saturated: 15.96%mol DPPS, 18.64%mol DPPC; unsaturated: 32.42%mol DOPC and 32.98%mol DOPE); for U-937: 29%mol Chol, 71%mol phospholipids (saturated: 12.80%mol DPPS, 30.70%mol DPPC; unsaturated: 23.80%mol DOPC and 32.70% mol DOPE). 

### 4.5. Langmuir Technique

The isotherms of the surface pressure (π) of monolayers as a function of the area per lipid molecule were obtained by using the Langmuir through (Minitrough, KSV, Finland) equipped with a Pt-Wilhelmy plate. Single-lipids and mixtures solutions were applied onto PBS subphase and left for 15 min according to the measurement procedure described previously [67,68,69]. 3FTx were spread with the Hamilton microsyringe (± 50μL)) at the established concentrations. All experiments were made at 25 °C (±1 °C). From surface pressure isotherms, physicochemical parameters of phospholipid monolayers were determined. These parameters are: (i) the pressure at which the layer collapsed π_coll_ (this parameter characterizes the state of the layer at the largest packing of the molecules), (ii) the limiting area per molecule representing maximal density of a layer (A_lim_) at the pressure that mimicking natural cell systems (π =30 m/Nm; A_30_), and (iii) the static compression modulus, representing mechanical resistance against layer compression to compare the state of monolayers:

$$C_{s}^{-1}=\;-\;\frac{d\pi }{d\ln A_{m}}$$

where A_m_ is the mean area per molecule and π is the surface pressure [70]. The excess free energy of mixing (based on the values obtained from mixed ingredients for HL-60 and U-937 model monolayers modified by 3FTx) was calculated, according to the formula of Costin and Barnes [71] and Flasiński et al. [72]:

$$\Delta G^{\mathrm{exc}}=N_{A}\int_{0}^{\pi }\left(A-A^{\mathrm{id}}\right)d\pi$$

The necessary values of the molecular areas of the components under ideal mixing conditions were obtained from the following equation:

$$A^{\mathrm{id}}=\sum A_{i}X_{i}$$

where A_i_ is mean area per molecule for the one component monolayer and X_i_ is the mole fraction of the lipid component in the mixed monolayer. 

### 4.6. Cell Cultures 

U-937 (Human histiocytic lymphoma cell line) and HL-60 (human promyelocytic cells line) (both purchased from ATCC) were cultured in suspension in RPMI 1640 (Cyto Gen GmbH); U-937 contained5% bovine serum (FBS), while HL-60 contained 10% FBS. A 0.01% penicillin-streptomycin mixture was applied to both cultures. After incubation at 37 °C/24 h in a humidified atmosphere, cells were used for the experiments.

### 4.7. Cell Viability Assay 

To evaluate cell viability, the XTT assay kit (Abcam; ab232856) was used according to the manufacturer’s instructions. Cells were cultured in 96-well plates at an amount of 0.2 × 10^6^ cells per well in a volume of 0.2 mL/well. After 24 h of exposing the cells to the 3FTx fraction, the reaction mixture was added for 2 h. Absorbance of the supernatant was read on a microplate reader (Epoch BioTek Instruments microplate reader, Winooski, VT, USA) at 450 nm. 

Based on the results of the cell survival presented in this paper, the values of the median lethal dose (LD50 median lethal dose) for cells were calculated using Behrens’ method [32] with the following assumptions: the number of cells per measurement point is constant, the absorbance determined in the XTT method is correlated with the number of live cells; moreover, if the cell is experiencing a higher dose, it has survived all lower doses; if the cell dies at a lower dose, it would die at all higher doses. Then, the percentage mortality was calculated for each dose of applied venom fraction and LD50 values were obtained from the appropriate graphs. 

### 4.8. Membrane Damage Assay (LDH Assay)

Lactate dehydrogenase leakage (LDH assay) was used for determination of the membrane stability after the 3FTx treatment. In the case, 100 μL of the supernatants obtained after the centrifugation was added to the mixture of 0.5 mL 0.75 mM sodium pyruvate and 10 μL of 140 μM NADH. After incubation over 30 min at 37 °C, 0.5 mL of 0.1 M 2,4-dinitrophenylhydrazine was added to each of the samples. The absorbance of formed hydrazone was measured spectrophotometrically at 450 nm after 1 h using the microplate reader Epoch (BioTek Instruments). The amount of LDH released due to complete disruption of the cell membrane by sonification was taken as a control.

### 4.9. Determination of Membrane Lipid Peroxidation (MDA Concentration)

Membrane lipid peroxidation was assessed spectrophotometrically measuring absorbance of thiobarbituric acid and malodialdehyde (MDA) complex at 532 nm. Cells were cultured in 24-well plates with 1 × 10^6^ cells per well with a volume of 0.5 mL/well. Next, they were treated with selected factors, adjusted to a final volume of suspension equal to 0.5 mL, and kept for 24 h. To the obtained supernatants, 0.5 mL of 0.5% trichloroacetic acid (TCA) was added. Then, the mixtures were vortexed for 1 min and then lysed over 5 min using an ultrasonic bath (15 kHz). The samples were centrifuged (10 min, 10,000× *g*) and finally added to 1.25 mL of solution of 20% TCA and 0.5% TBA. After 30 min of heating at 100 °C in a dry thermoblock, the samples were cooled down. Membrane lipid peroxidation was assessed photometrically, measuring the absorbance of malondialdehyde (MDA) at 532 nm (corrected for non-specific background by subtracting the absorbance at λ = 600 nm), using the molar extinction coefficient of MDA equal to 155 $\frac{1}{mM*cm}$M.

### 4.10. Nitric Oxide Production 

Cells (cultured in the amount of 2 × 10^6^ per well) were treated with venom fraction, adjusted to a final volume of suspension equal to 0.5 mL, and kept for 24 h. After treatment, the supernatants were collected, centrifuged (1000× *g*, 5 min), and stored at –20 °C. Nitric oxide (NO_x_) production from treated cells was quantified spectrophotometrically using the Griess reagent (modified) (Sigma–Aldrich, Minch, Germany). The absorbance was measured at 540 nm and the nitrite concentration was determined using the calibration curve. 

### 4.11. Statistical Analysis

In the biochemical experiments (LDH, MDA, cell viability, NO concentration), measurements were repeated at least three times, and each experiment included at least three to five individual treatments for different concentrations of 3FTx (from 0–120 ng/mL). 

The physicochemical experiments were repeated three to five times to ensure a high reproducibility of the obtained isotherms to ±0.1–0.3 Å^2^ (obtained by KSV NIMA software). The accuracy of the surface tension measurements was ±0.1 mN/m. On the basis on isotherms, parameters (A_lim_, π_coll_, C_s_^−1^) were calculated, according to the procedure described in the ‘Materials and Method’ (Section 4.5) using the program SigmaPlot. 

For the obtained data, the standard deviation, which determines the amount of dispersion away from the mean, was calculated. Data from the various treatments were statistically analyzed using Duncan’s multiple range test from PC SAS 8.0 software. Differences of *p* ≤ 0.05 were considered to be significant.

The scheme of describing statistically significant differences was standardized for all Tables and Figures. Significant differences between 3FTx concentrations within a U-937 cell line were marked by uppercase letters, and for the HL-60 cell line, lowercase letters were selected; asterisks characterize the difference between the tested model membranes, mimicking the U-937 and HL-60 cell lines. Statistically significant differences between the tested lipids (single-component monolayers) were marked: for the 10 ng/mL concentration with Roman numerals; for the 40 ng/mL concentration with the letters of the Greek alphabet.

## 5. Conclusions

Based on the conducted experiments, it was shown that one of the mechanisms of action of the 3FTx protein family is their interaction with the lipid part of the cell membrane. The results clearly show that the level of these interactions depends on electrostatic interactions with the hydrophilic part (decisive at higher concentrations of toxins at higher concentrations), and is also related to the degree of saturation of fatty acids in hydrophobic parts of lipids (observed at lower concentrations of 3FTx). The greatest interaction of 3FTx with the polar part was demonstrated for zwitterionic lipids and the lowest for negatively charged lipids. Therefore, it is suggested that membranes that consist of these lipids, greater cholesterol content and more unsaturated fatty acids in their structure, will be more susceptible to interaction with 3FTx. The above construction features of the membrane characterize the tested HL-60 line. Compared to the U-937 membrane, the HL-60 immune cell showed greater membrane damage, lower viability, and greater sensitivity due to the 3FTx dose (according to LD50). In studies of physiological and biochemical parameters, it was unequivocally found that the toxic effect of 3FTx on the membrane level consequently influences the physiology of the whole cell.

## Figures and Tables

**Table 1:** Effect of four different 3FTx concentrations on parameters characterizing monolayers of model membrane systems. Values represent the average ± SD (*n* = 5). Significant differences (*p* ≤ 0.05) between model membranes systems are marked with an asterisk and between different 3FTx concentration: for U-937 cell line-uppercase letters, for HL-60 cell line-lowercase letters.

Experiment	A_lim_ (Å^2^)		π_col_ (mN/m)		C_s_^−1^_max_ (mN/m)	
	U-937	HL-60	U-937	HL-60	U-937	HL-60
Control	54.2 ± 0.1 ^E,^*	52.9 ± 0.1 ^d^	44.0 ± 0.9 ^A^	45.9 ± 0.5 ^a^	149.4 ± 0.1 ^A^	170.6 ± 0.1 ^a,^*
5 ng/mL	57.3 ± 0.2 ^D,^*	56.4 ± 0.1 ^c^	44.7 ± 0.8 ^A^	42.5 ± 0.8 ^b^	130.4 ± 0.2 ^B^	133.4 ± 0.1 ^b,^*
10 ng/mL	58.6 ± 0.2 ^C^	58.6 ± 0.3 ^b^	42.3 ± 0.8 ^B^	42.3 ± 0.6 ^b^	112.4 ± 0.3 ^C^	113.0 ± 0.2 ^c,^*
20 ng/mL	59.1 ± 0.1 ^B,^*	58.8 ± 0.1 ^b^	41.9 ± 0.6 ^B^	42.2 ± 0.5 ^b^	94.3 ± 0.3 ^D^	93.9 ± 0.2 ^d^
40 ng/mL	60.2 ± 0.1 ^A^	61.1 ± 0.1 ^a,^*	44.5 ± 0.8 ^A^	42.4 ± 0.6 ^b^	89.9 ± 0.3 ^E^	89.6 ± 0.3 ^e^

**Table 2:** Values of the medial lethal dose, calculated for the HL-60 and U-937 tested cells. Values represent the average ±SD (n = 5). The asterisk indicates a significant difference between the tested cell lines (*p* ≤ 0.05).

Cell line	LD50 [mg/L/1 × 10^6^ cells]
U-937	126.80 ± 2.94 *
HL-60	121.29 ± 1.42

## Data Availability

Not applicable.

## Supplementary Materials

Table S1:Complete list of all identified proteins in Fraction G with its quantitation analysis, Figure S2a,b: Effect of 3FTx on (a) the limiting area per molecule A_30_ (obtained at π = 30) and (b) on static compression modulus(C_s_^−1^) obtained for the HL-60 and U-937 model membranes, Figure S3a–f: Surface pressure isotherms (π) as a function of the area per lipid molecule and the dependencies of the static compression modulus (C_s_^−1^) vs. the surface pressure (π) (in the corner) for phosphatidylethanolamine (DOPE) and phosphatidylserine (DPPS), phosphatidylcholine (16:0)(DPPC)and phosphatidylcholine (18:1) (DOPC), and cholesterol (Chol), Figure S4: Level of NO_x_ secreted by HL-60 and U-937 tested cells after contact with 3FTx.

## Sample Availability

Not available

## Abbreviations

A_lim_	the limiting area per molecule representing the maximal density of a layer
A_30_	the area per molecule representing the density of a layer at π = 30 mN/m
3FTx	three-finger toxins
C_s_^−1^	static compression modulus
∆G^exc^	the excess free energy of mixing
DPPC	1,2-dipalmitoyl-sn-glycero-3-phosphocholine (16:0)
DOPC	1,2-dioleoyl-sn-glycero-3-phosphocholine (18:1)
DOPE	1,2-dioleoyl-sn-glycero-3-phosphoethanolamine (18:1)
DPPS	1,2-dipalmitoyl-sn-glycero-3-phospho-L-serine (16:0)
Chol	cholesterol
MDA	malondialdehyde
LDH	lactate dehydrogenase
LD50	medial lethal dose
NO	nitric oxide
