# Supplementary material for: Effects of 3FTx Protein Fraction from Naja ashei Venom on the Model and Native Membranes: Recognition and Implications for the Mechanisms of Toxicity
Source: Molecules. 2021 Apr 9;26(8):2164. doi: 10.3390/molecules26082164 (PMC8070352; doi:10.3390/molecules26082164)
Supplement: Supplementary file 1 [file molecules-26-02164-s001.zip › molecules-1139522-supplementary.pdf]

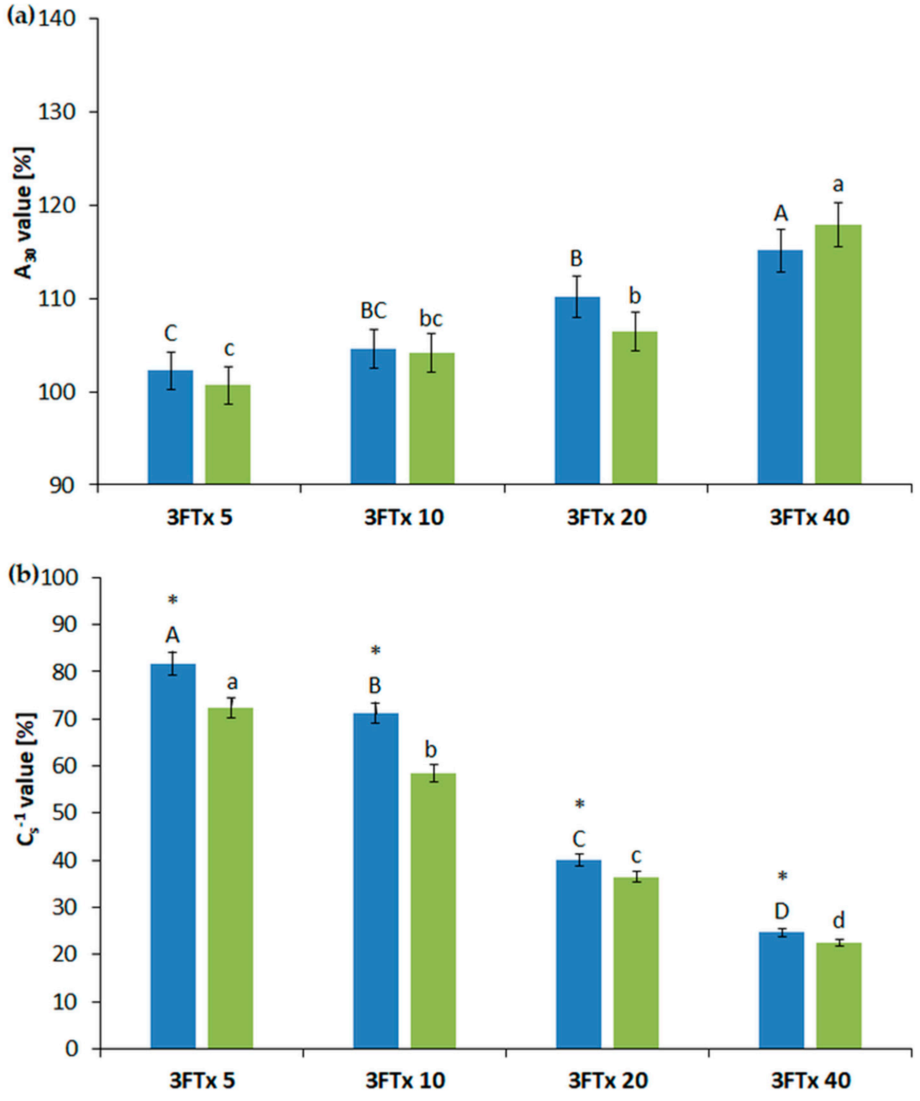

Figure S2. Effect of 3FTx on **a)** the limiting area (measured for π=30) and on **b)** static compression modulus (C_s^{-1}) for model HL-60 (green colour) and model U-937 (blue colour). Values represent the average ± SD (n=5). Significant differences were marked: between 3FTx concentrations (3FTx 5 - 5ng/ml, 3FTx 10 - 10ng/ml, 3FTx 20 - 20ng/ml, 3FTx 40 - 40ng/ml) by different letters: within a U-937 cell line were marked by uppercase letters and for HL-60 cell line - lowercase letters; between tested models (for a given concentration) by an asterisk ($p \leq 0.05$).

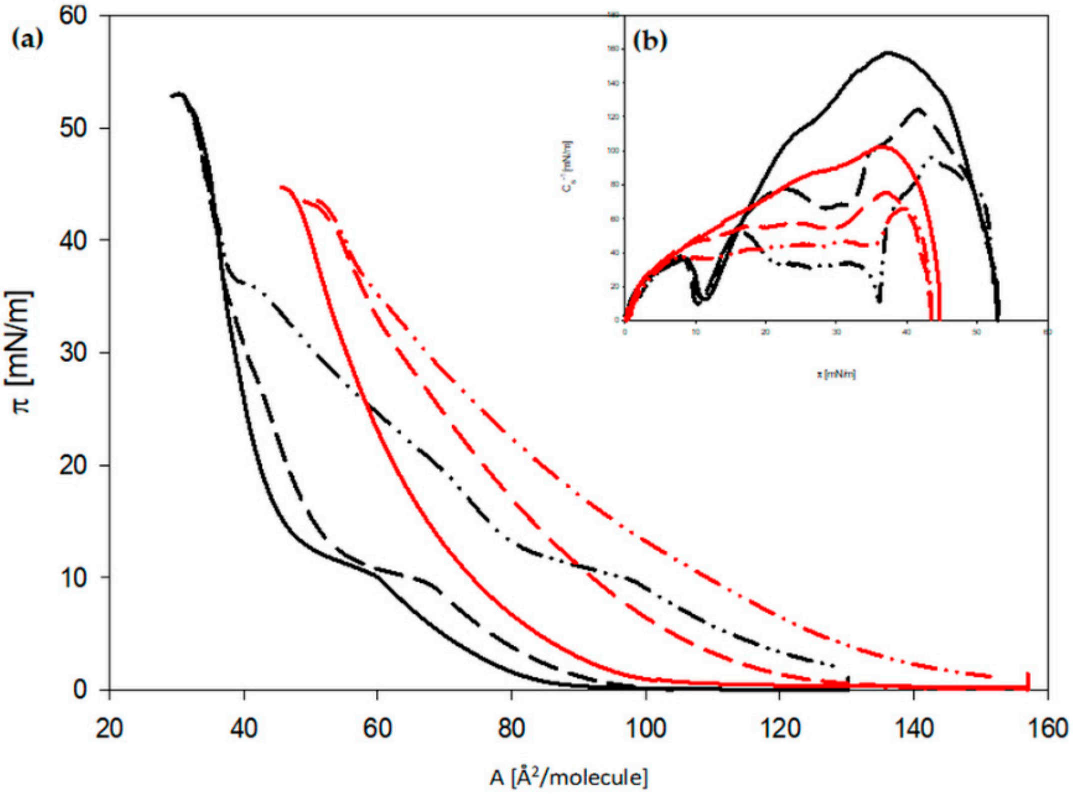

Figure S3. (a) Surface pressure isotherms (π) as a function of the area per lipid molecule and **(b)** the dependencies of static compression modulus (C_s^{-1}) vs. π for phosphatidylcholines (DOPC (18:1)- red lines and DPPC (16:0) – dark lines). Lipids were spread on phosphate buffer – solid lines, on phosphate buffer with 10ng/ml 3FTx– short line and on phosphate buffer with 40ng/ml 3FTx– double dotted – dashed lines.

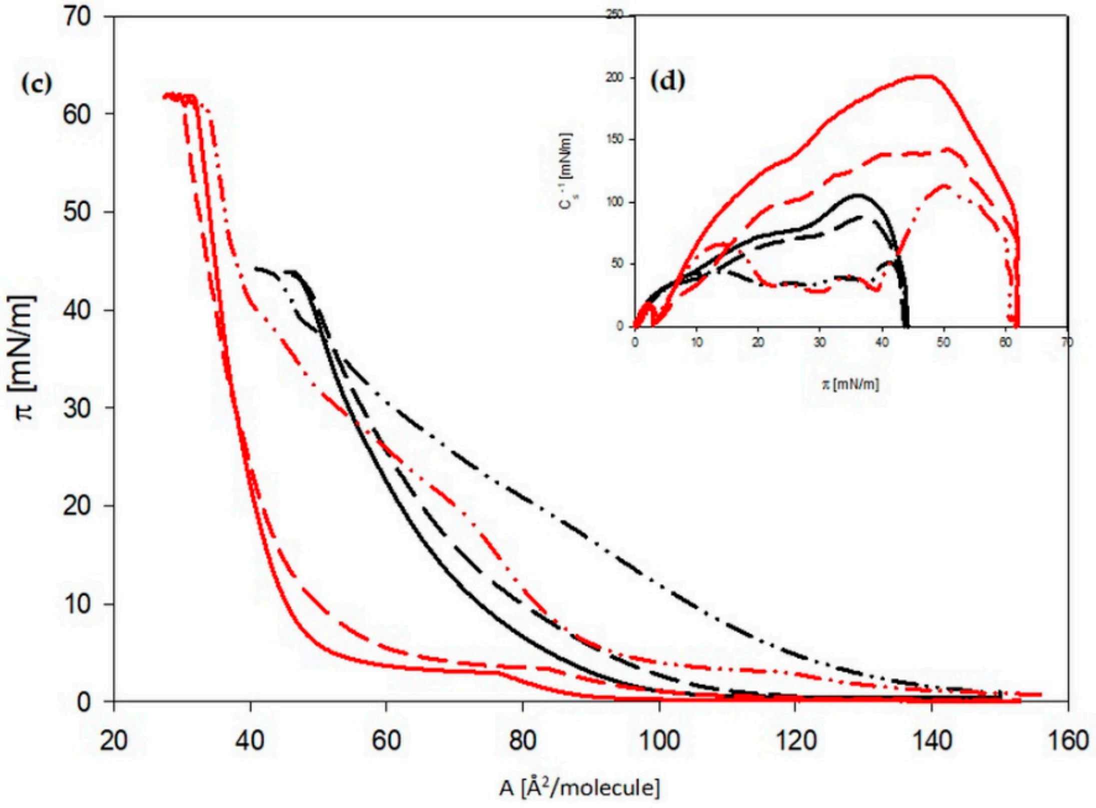

Figure S3. (c) Surface pressure isotherms (π) as a function of the area per lipid molecule and **(d)** the dependencies of static compression modulus (C_s^{-1}) vs. π for phosphatidylserine (DPPS (16:0) - red lines) and DOPE (18:1) – dark lines). Lipids were spread on phosphate buffer – solid lines, on phosphate buffer with 10ng/ml 3FTx– short lines and on phosphate buffer with 40ng/ml 3FTx– double dotted – dashed lines.

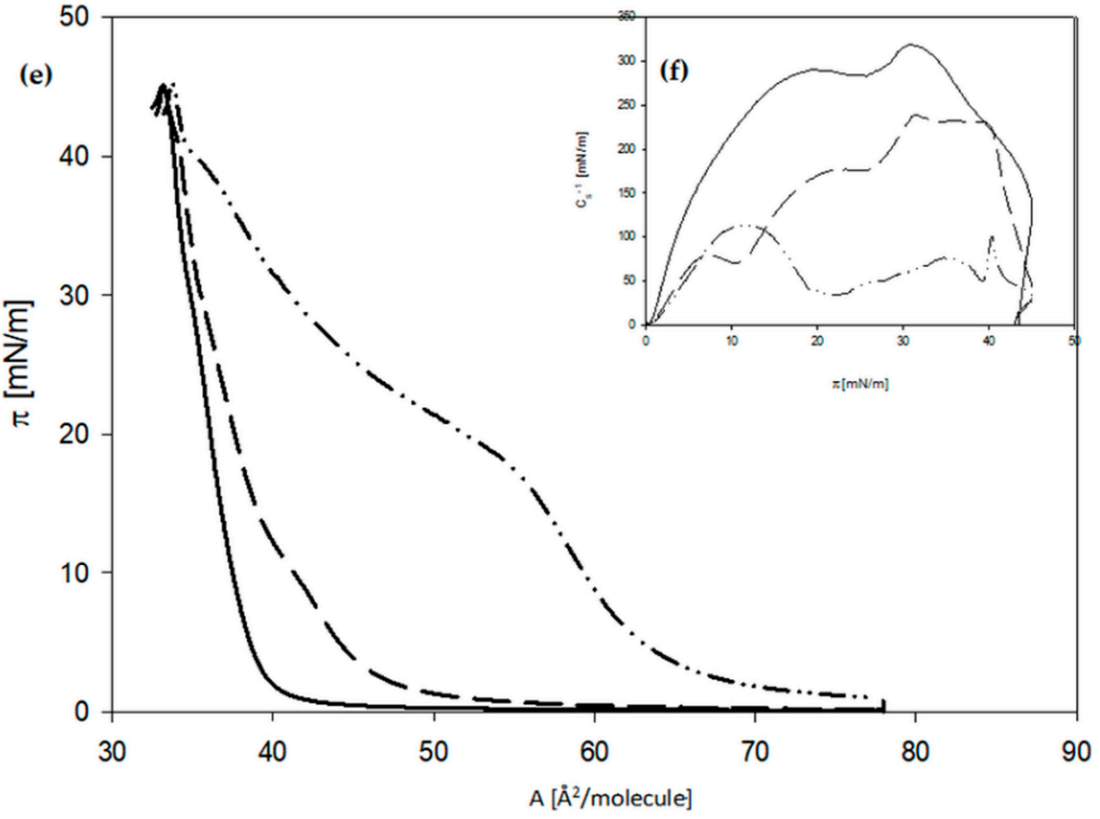

Figure S3. (e) Surface pressure isotherms (π) as a function of the area per lipid molecule and **(f)** the dependencies of static compression modulus (C_s^{-1}) vs. π for cholesterol. Lipid were spread on phosphate buffer – solid line, on phosphate buffer with 10ng/ml 3FTx– short line and on phosphate buffer with 40ng/ml 3FTx– double dotted – dashed line.

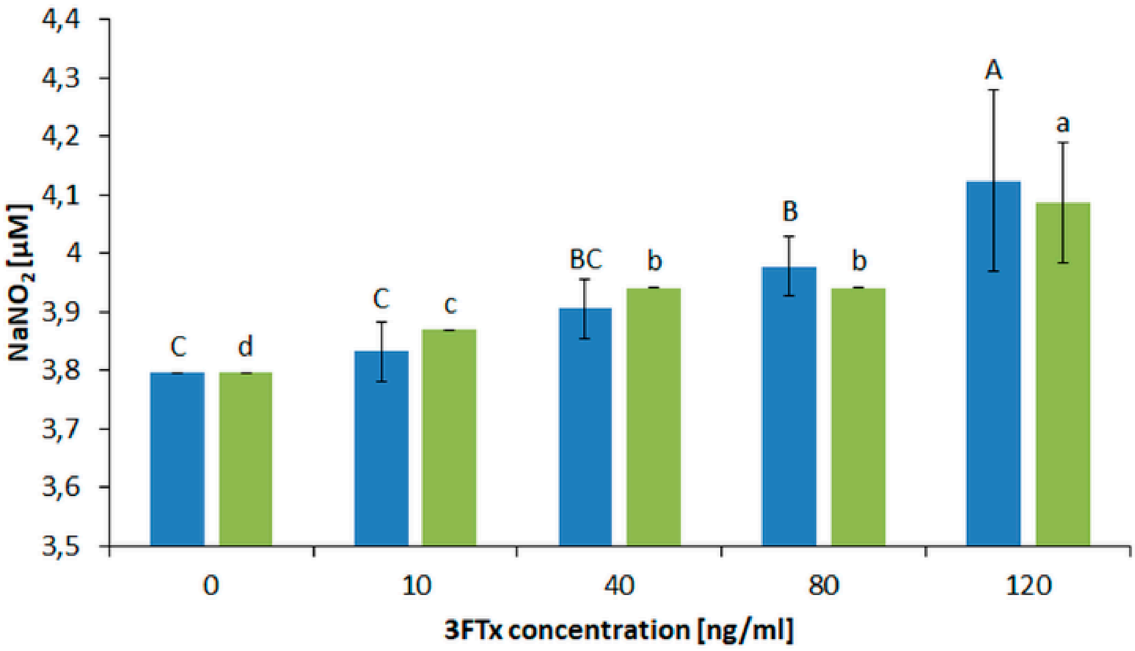

Figure S4: Level of NOx secreted by HL-60 (green colour) and U-937 (blue colour) after contact with 3FTx.

NO_x production was quantified spectrophotometrically using the Griess reagent. Cells were treated with the indicated concentrations for 24 h. Data points are means ± SD (n=5) from a representative experiment. Statistically significant differences were marked: between 3FTx concentrations (3FTx 5 - 5ng/ml, 3FTx 10 - 10ng/ml, 3FTx 20 - 20ng/ml, 3FTx 40 - 40ng/ml) by different letters: within a U-937 cell line were marked by uppercase letters and for HL-60 cell line - lowercase letters; between tested models (for a given concentration) by an asterisk ($p \leq 0.05$).
